# A Novel Dominantly Segregating PSMB10 Splice-Site Variant in Familial Autoinflammatory Disease with Immunoproteasome and Interferon-Related Dysregulation

**DOI:** 10.3390/genes17090997

**Published:** 2026-08-25

**Authors:** Umut Inci Onat, Alper Bülbül, Dora Sigli, Elif Arık Sever, Serdal Ugurlu, Ayse Huri Ozdogan, Eda Tahir Turanli

**Affiliations:** 1 Department of Molecular Biology and Genetics, Faculty of Engineering and Natural Sciences, Acibadem Mehmet Ali Aydinlar University, 34752 Istanbul, Türkiye; inci.onat@acibadem.edu.tr (U.I.O.); siglidora@gmail.com (D.S.); 2 Department of Molecular Biology and Genetics, Graduate School of Natural and Applied Sciences, Acibadem Mehmet Ali Aydinlar University, 34752 Istanbul, Türkiye; 3 Department of Biostatistics and Bioinformatics, Graduate School of Health Sciences, Acibadem Mehmet Ali Aydinlar University, 34752 Istanbul, Türkiye; alper.bulbul1@gmail.com (A.B.); arikelif@gmail.com (E.A.S.); 4 Izmir Biomedicine and Genome Center (IBG), 35340 Izmir, Türkiye; 5 Izmir International Biomedicine and Genome Institute, Dokuz Eylul University, 35340 Izmir, Türkiye; 6 Department of Biostatistics and Medical Informatics, School of Medicine, Bahcesehir University, 34353 Istanbul, Türkiye; 7 Department of Biomedical Engineering, Graduate School of Natural and Applied Sciences, Acibadem Mehmet Ali Aydinlar University, 34752 Istanbul, Türkiye; 8 Division of Rheumatology, Department of Internal Medicine, Cerrahpasa Medical Faculty, Istanbul University-Cerrahpasa, 34098 Istanbul, Türkiye; serdalugurlu@gmail.com (S.U.); huriozdogan@yahoo.com (A.H.O.); 9 Medica Medical Center, 34367 Istanbul, Türkiye

**Keywords:** exome sequencing, RNA splice sites, hereditary autoinflammatory diseases, interferon type I, gene expression, family

## Abstract

Background: Systemic autoinflammatory phenotypes can clinically overlap with Familial Mediterranean Fever and other monogenic autoinflammatory diseases, yet some cases remain unclassified in the absence of pathogenic variants in *MEFV* or other related genes. In this study, we aimed to investigate the genetic and molecular basis of an unclassified autoinflammatory phenotype in a two-generation family comprising four affected members and one unaffected member. Methods: We performed whole-exome sequencing in all family members and prioritized variants according to rarity, predicted functional impact, segregation pattern, and biological relevance to inflammatory pathways. Downstream molecular analyses were performed using PBMCs from affected individuals and the unaffected family member. Results: Whole-exome sequencing identified a novel splice-site variant in *PSMB10* (NM_002801; c.56+1G>A) affecting the canonical splice donor site. The variant segregated with the autoinflammatory phenotype and was associated with reduced full-length *PSMB10* transcript levels in patient-derived PBMCs, supporting a predicted loss-of-function effect. Although pathogenic variants in *PSMB10* have previously been implicated in proteasome-associated autoinflammatory syndrome (PRAAS), the clinical presentation in this family differed from the classical PRAAS phenotype. Molecular analyses showed altered immunoproteasome-related gene expression and a severity-associated interferon-related response. Severely affected individuals showed increased expression of interferon-related genes, including *ISG15*, *IFI35*, and *SIGLEC1*, whereas mildly affected individuals showed lower or reduced expression patterns. Notably, the extent of these molecular alterations differed among family members and broadly reflected the observed clinical heterogeneity. Conclusions: We report a novel *PSMB10* splice-site variant as a strong potential contributor to an unclassified autoinflammatory disease, with a predicted disruption of canonical splicing and loss of protein function. Our findings expand the clinical spectrum of immunoproteasome-associated disorders and suggest that immunoproteasome-related dysregulation and variable interferon responses may contribute to disease severity. The intrafamilial variability observed in this family suggests that additional genetic or immunogenetic factors may modify disease expression, even in autoinflammatory disorders that appear to follow a monogenic inheritance pattern.

## 1. Introduction

Genetic autoinflammatory diseases are characterized by recurrent episodes of fever and systemic inflammation, often accompanied by serositis, rash, urticaria, lymphadenopathy, arthritis, and elevated acute-phase responses. These disorders primarily result from inherited defects in innate immune regulation. In contrast to autoimmune diseases, autoinflammatory conditions occur in the absence of disease-specific autoantibodies or autoreactive T cells. Although most monogenic autoinflammatory diseases present during childhood, delayed or adult-onset presentations may also occur [1]. Familial Mediterranean Fever (FMF) is the prototypic and most common monogenic autoinflammatory disease [2,3], particularly in populations of Mediterranean origin. FMF is not restricted to the Turkish population; it is also common among Armenian, Arab, and Jewish communities [4]. It has been reported, although less frequently, in Italian cohorts [5], reflecting the shared genetic ancestry and geographic distribution of *MEFV* founder variants across the Mediterranean basin [3]. Other monogenic autoinflammatory diseases include Tumor Necrosis Factor Receptor-Associated Periodic Syndrome (TRAPS), Mevalonate Kinase Deficiency/Hyperimmunoglobulinemia D Syndrome (MKD/HIDS), Cryopyrin-Associated Periodic Syndromes (CAPS), Blau syndrome, Deficiency of the Interleukin-1 Receptor Antagonist (DIRA), Deficiency of the Interleukin-36 Receptor Antagonist (DITRA), Pyogenic Arthritis, Pyoderma gangrenosum, and Acne syndrome (PAPA), Majeed syndrome, and Chronic Atypical Neutrophilic Dermatosis with Lipodystrophy and Elevated Temperature/Proteasome-Associated Autoinflammatory Syndrome (CANDLE/PRAAS) represent less frequent entities within this spectrum [6,7,8].

A substantial proportion of patients with suspected systemic autoinflammatory diseases lack identifiable pathogenic variants in known disease genes, which may reflect overlapping clinical presentations, atypical or adult-onset disease, incomplete genomic assessment, and the contribution of yet-unidentified genes or modifier mechanisms [9,10,11,12]. In some instances, diagnostic certainty is supported by the integration of genetic findings, while the clarification of underlying molecular pathways facilitates the optimization of therapeutic strategies [13].

In regions with high FMF prevalence, founder effects, consanguinity, and population-specific genetic heterogeneity may complicate both clinical and molecular diagnosis [14,15]. FMF-like phenotypes without biallelic pathogenic *MEFV* variants may reflect overlap syndromes or alternative monogenic etiologies, highlighting the need for broader molecular evaluation in unresolved cases [16]. In this context, *PSMB10* has emerged as a relevant candidate gene at the interface between proteasome-associated autoinflammation and Familial Mediterranean Fever (FMF)-like phenotypic overlap [17].

*PSMB10* encodes the immunoproteasome β2i subunit, and pathogenic variants in this gene have been linked to proteasome-associated autoinflammatory syndromes [17,18]. Disruption of *PSMB10* function may compromise immunoproteasome maturation and proteolytic activity, leading to impaired proteostasis, accumulation of proteotoxic substrates, and dysregulated type I interferon-related innate immune signaling [18,19]. Cellular protein homeostasis and innate immune signaling therefore depend heavily on the precision of the ubiquitin–proteasome system [19].

Proteasome-associated autoinflammatory diseases have been linked to impaired protein clearance, immune dysregulation, and type I interferon-related inflammatory responses [18,20]. These observations provide a rationale for evaluating immunoproteasome regulation and selected IFN-related molecular responses in unresolved autoinflammatory phenotypes.

In this study, we used whole-exome sequencing to investigate a family with multiple cases of undiagnosed autoinflammatory disease. The clinical presentation showed overlap with FMF and other monogenic autoinflammatory disorders; however, the affected individuals lacked pathogenic variants in established autoinflammatory genes, including *MEFV*, *NLRP3*, *TNFRSF1A*, *MVK*, *NOD2*, *NLRC4*, *PSTPIP1*, *LPIN2*, *IL1RN*, *IL36RN*, *TNFAIP3*, *NLRP12*, and *PLCG2*. The affected individuals also showed inadequate responses to conventional treatment. We identified the *PSMB10* variant c.56+1G>A (rs201451622) and evaluated its predicted effect on disruption of the canonical splice donor site, transcript-level expression, together with immunoproteasome and selected IFN-related molecular profiles in affected family members. Among the IFN-related markers evaluated, the most notable differences were observed in *SIGLEC1*, *IFI35*, and *ISG15*, particularly in the more severely affected individuals.

## 2. Materials and Methods

### 2.1. Participants and Study Approval

A non-consanguineous Turkish family with suspected autosomal dominant inheritance, comprising three affected siblings, their affected father, and an unaffected mother, was enrolled in the study. All affected family members were followed at Istanbul University-Cerrahpasa for suspected autoinflammatory disease. Written informed consent was obtained from all participants prior to enrollment. All study procedures were conducted in accordance with the ethical principles of the institutional research committee and the 1964 Declaration of Helsinki, including its subsequent amendments. The study protocol was approved by the Health and Engineering Sciences Human Research Ethics Committee of Istanbul Technical University (approval no. MBG-2/2017).

### 2.2. Sample Collection and Preparation

Genomic DNA was extracted from whole blood samples using the Mammalian Blood DNA Isolation Kit (Roche Diagnostics, Mannheim, Germany) according to the manufacturer’s instructions. The remaining blood samples were processed for whole-blood RNA extraction and peripheral blood mononuclear cell (PBMC) isolation. For PBMC isolation, 20 mL of venous blood was subjected to density-gradient centrifugation using Lymphocyte Separation Medium (Capricorn Scientific, Ebsdorfergrund, Germany).

### 2.3. Whole Exome Sequencing and Analysis

Whole exome sequencing (WES) was performed for the four affected patients and the unaffected relative using the Twist Human Core Exome and RefSeq kit on the Illumina platform with paired-end reads (Macrogen Europe, Amsterdam, The Netherlands). Raw paired-end reads were aligned to the GRCh38 human reference assembly with BWA-MEM v0.7.17 [21] using default parameters; the resulting alignments were coordinate-sorted, and PCR duplicates were flagged and removed with samtools markdup v1.19 [22]. The deduplicated, coordinate-sorted BAM files were per-sample variant-called with DeepVariant v1.5.0 [23] and annotated with OpenCRAVAT 3.1.1 [24], integrating CADD v1.6 [25], gnomAD v3.1.2 [26], ClinVar, and Human Phenotype Ontology mappings [27].

HLA class I (HLA-A, -B, -C, and the non-classical -E, -F, -G) and class II (HLA-DRB1, -DRB3/4/5, -DQA1, -DQB1, -DPA1, -DPB1) typing was performed directly from paired-end whole-exome FASTQ reads using HLA-HD v1.7.1 [28] against the IPD-IMGT/HLA reference database, returning 4-digit (2-field) allele assignments per locus.

### 2.4. In Silico Splice Site Analysis

The transcript-level consequences of *PSMB10* NM_002801.3: c.56+1G>A were predicted using SpliceAI v1.3.1 [29], Pangolin v1.0.1 [30], and SAI-10k-calc v1.1 [31]. The intrinsic strength of the affected splice site was quantified using MaxEntScan v1.1, a maximum-entropy model of human splice-site sequences that accounts for dependencies between both adjacent and non-adjacent positions and returns a log-odds score in bits [32].

### 2.5. RNA Isolation and Quantitative Real-Time PCR Analysis

Total RNA was extracted from whole blood with Trizol Reagent (Thermo Fisher Scientific, Waltham, MA, USA) and cDNA was synthesized from 1 µg of total RNA using the High-Capacity cDNA Reverse Transcription Kit (Applied Biosystems, Foster City, CA, USA) following the manufacturer’s instructions. qPCR was performed to assess gene expression for *PSMB10* Exon1/Exon2 Junction, *PSMB10*, *PSMB8*, *ISG15*, *IFI35* and *SIGLEC1*, and analyzed for relative gene expression by the 2^−∆∆Ct^ method [33].

Two complementary *PSMB10* qRT-PCR assays were used. The exon 1–exon 2 assay was designed to quantify the canonical exon 1–exon 2 splice product, whereas the total *PSMB10* assay targeted a shared exonic region and was used as a broader measure of *PSMB10* transcript abundance. Accordingly, the exon 1–exon 2 assay was used to assess the abundance of the normally spliced transcript rather than to directly characterize the identity of any aberrant splice product.

Primers used in this study are given below:
**Primer Name****Direction****Sequence 5′→3′**hPSMB10ForwardCCTGGCGGTGCTAGAAGAChPSMB10ReverseGATCACACATGCGTCCACATThPSMB8ForwardGCAGGCTGTACTATCTGCGAAhPSMB8ReverseAGAGCCGAGTCCCATGTTCAThISG15ForwardCGCAGATCACCCAGAAGATCGhISG15ReverseTTCGTCGCATTTGTCCACCAhIFI35ForwardAACAAAAGGAGCACACGATCAhIFI35ReverseCTCCGTTCCTAGTCTTGCCAAhSIGLEC1ForwardATGGGGTACGCCTCCAAAChSIGLEC1ReverseGTGCCTCATTGGGTGTGTTGhGAPDHForwardTGCACCACCAACTGCTTAGChGAPDHReverseGGCATGGACTGTGGTCATGAGPSMB10-Exon-1/2 JunctionForwardCTGCCAAAGAAATGCATCATTGPSMB10-Exon-1/2 JunctionReverseGACGCGTTCCAATGATGCATT

### 2.6. Statistics

Gene expression measurements were obtained from a single biological sample per individual (four samples as independent healthy controls and one sample per family member). Each qPCR assay was performed in duplicate (two technical replicates). For each gene analyzed, the expression level of every family member was compared individually with the control baseline using nested *t*-tests or one-way ANOVA, as indicated in the figure legends, in GraphPad Prism 10 software. In these analyses, individual subjects were treated as the experimental unit; the models therefore incorporate intra-assay technical variation (duplicates) across all samples and inter-individual biological variation among the control subjects. *p*-values < 0.05 were considered statistically significant. When appropriate, Dunn’s multiple-comparisons post hoc test was applied.

## 3. Results

### 3.1. Clinical Characterization of the Family

**Figure 1:**
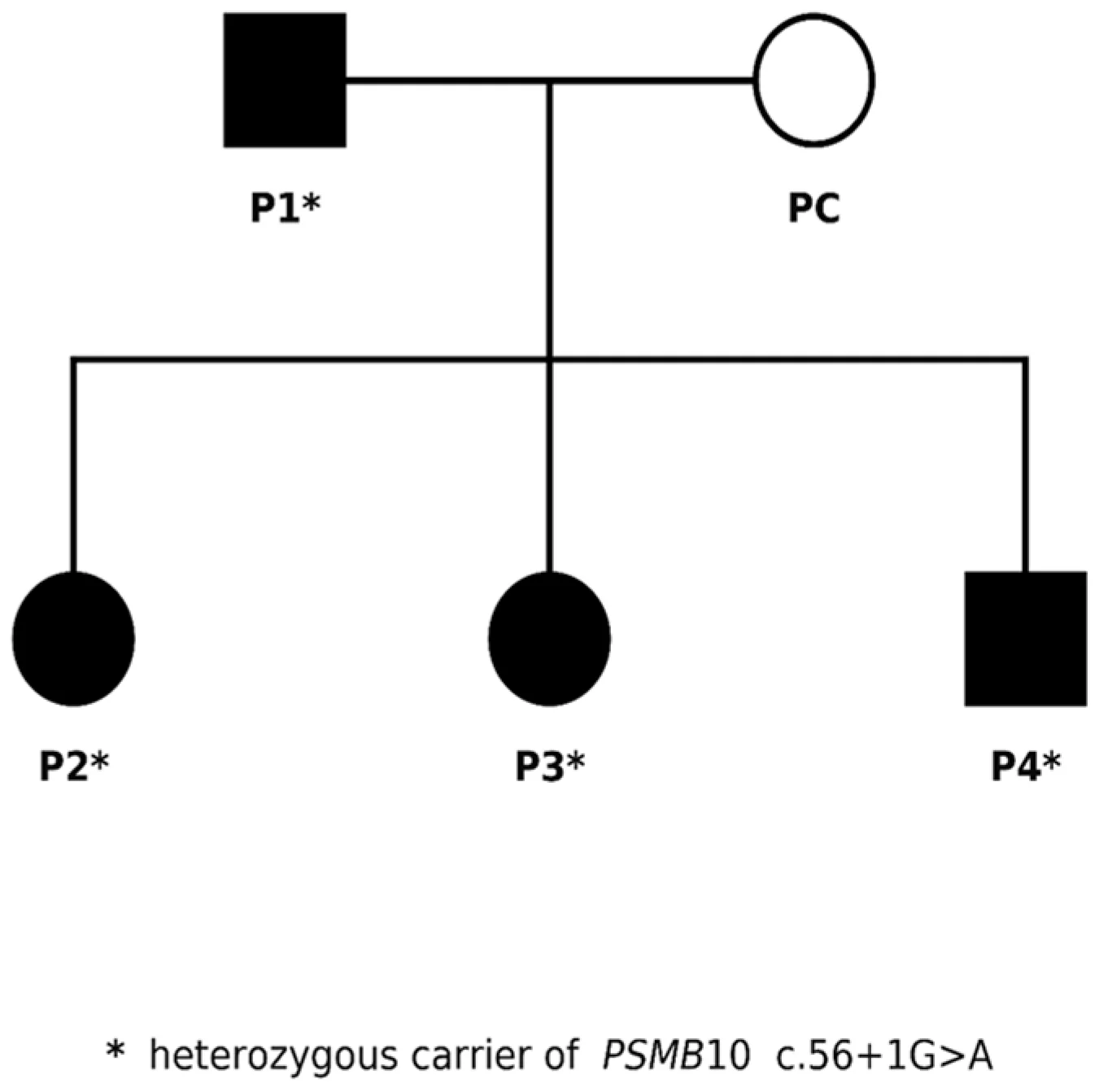
Pedigree of the Turkish family with four affected individuals showing a paternal segregation pattern. Squares = male, circles = female; filled = clinically affected, open = unaffected [34]. The asterisk (*) marks heterozygous carriers of *PSMB10* c.56+1G>A.

The study included a two-generation family with four clinically affected individuals and one unaffected family member. The pedigree in Figure 1 showed a dominantly segregating autoinflammatory phenotype through the paternal line. The affected father and all three affected offspring carried the *PSMB10* c.56+1G>A variant, whereas the unaffected mother was variant-negative.

Although all affected family members showed a multisystem inflammatory phenotype, the clinical presentation was heterogeneous within the family. The clinical characteristics of the five-member family are summarized in Table 1. The two affected siblings (P2 and P3) with detailed clinical data had childhood-onset disease characterized by recurrent fever, mucocutaneous findings, sicca symptoms, Raynaud phenomenon, arthritis/arthralgia, pleuritic chest pain, dyspnea, abdominal pain, and diarrhea. However, the severity and spectrum of organ involvement varied among affected individuals. Additional variable features included suspected uveitis, epilepsy, proteinuria, thyroid disease, recurrent ovarian torsion, congenital cataract, and severe weight loss in one affected sibling. The affected brother (P4) had a milder or less extensively documented phenotype, mainly characterized by intermittent abdominal pain and diarrhea, whereas the father (P1) had limited reported systemic manifestations. Prior clinical evaluations led to overlapping diagnoses, including FMF, juvenile/rheumatoid arthritis, Systemic Lupus Erythematosus (SLE), vasculitis/Henoch-Schonlein Purpura (HSP), and unclassified autoinflammatory disease. This familial clustering and intrafamilial clinical variability provided the rationale for performing whole-exome sequencing to identify shared candidate genetic variants segregating with the autoinflammatory phenotype.

### 3.2. Genetics: Identification of a Segregating PSMB10 Variant

**Figure 2:**
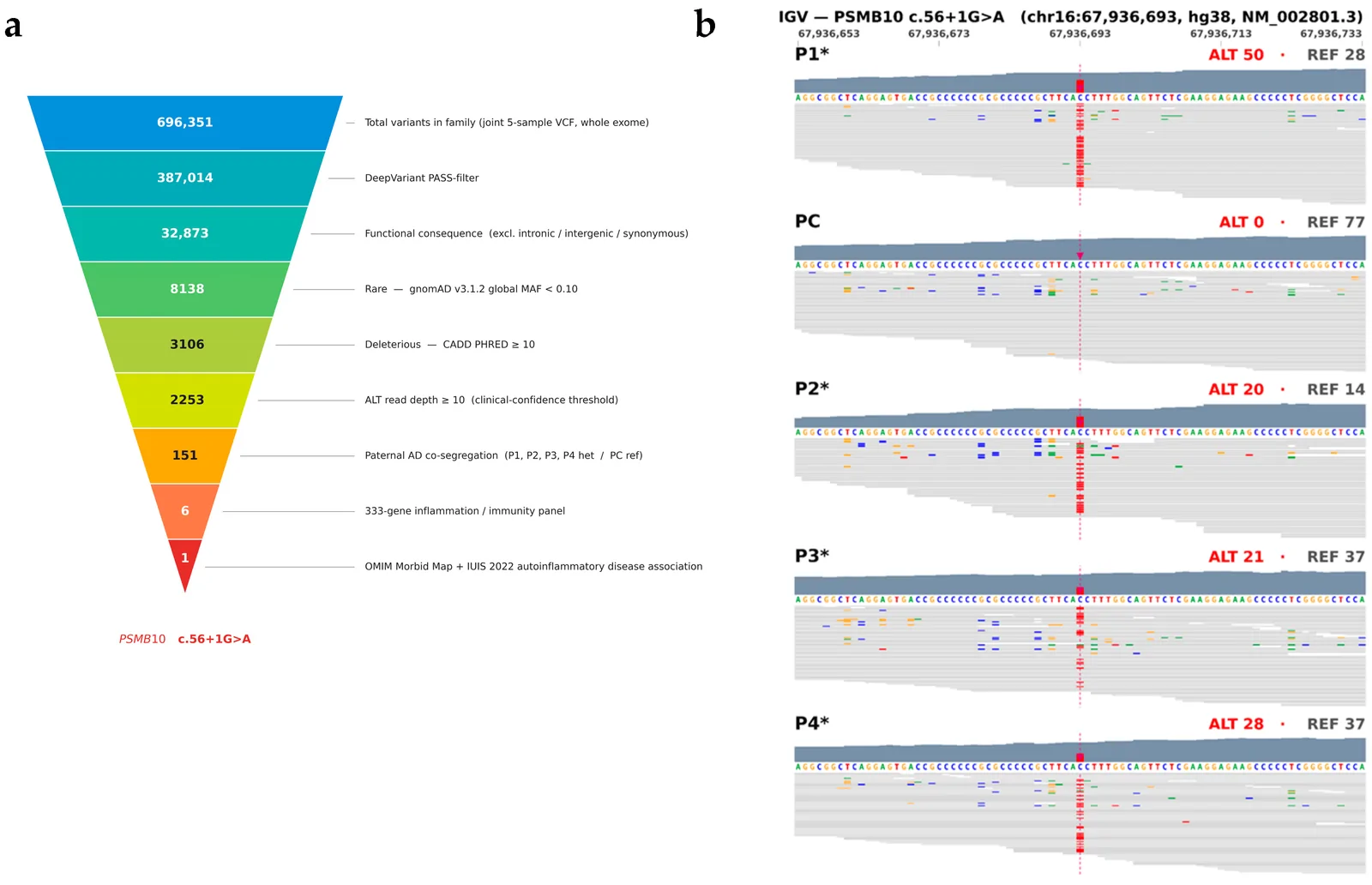
Variant identification and segregation patterns of *PSMB10* c.56+1G>A. (**a**) Variant filtering workflow for whole-exome sequencing analysis. Sequential filtering of the joint-called family whole-exome variant set identified a single candidate variant, *PSMB10* (NM_002801.3: c.56+1G>A), after quality, functional, population frequency, pathogenicity, segregation, gene panel, and disease annotation filtering. (**b**) Read-level evidence at the variant locus. IGV-style alignments at chr16:67,936,693 (BWA-MEM v0.7.17, samtools markdup v1.19) show heterozygous carriage in P1*, P2*, P3* and P4*, whereas PC shows reference-only reads, supporting paternal autosomal-dominant transmission. The red dashed line marks the variant column; alternate allele reads are shown in grey.

Whole-exome sequencing generated 696,351 distinct variants. After sequential filtering for high-confidence DeepVariant PASS calls, functional consequences, rarity, predicted deleteriousness, adequate read depth, and compatibility with an autosomal-dominant inheritance model, six candidate loci remained within the curated inflammation/immunity gene panel. Cross-referencing these candidates with OMIM Morbid Map and the IUIS inborn errors of immunity classification identified *PSMB10* as the only gene with an established autoinflammatory disease association (Figure 2a). The qualifying variant was a heterozygous splice-donor variant, *PSMB10* NM_002801.3: c.56+1G>A (rs201451622), with a high predicted deleteriousness score (CADD PHRED 34.0) and low population frequency in gnomAD v3.1.2 (global allele frequency 2.80 × 10^−4^). IGV-style alignments of each patient at the variant locus (chr16:67,936,693) (Figure 2b).

Further analysis for HLA is summarized in Table 2 and identified a paternal HLA haplotype that was shared by the affected father and all three affected children but absent in the unaffected mother. Five alleles showed this strict segregation pattern: HLA-B*44:02, HLA-C*16:04, HLA-G*01:04, HLA-DPB1*02:01, and HLA-DMA*01:02. Additional paternal alleles, including HLA-B*35:01, HLA-C*04:01, HLA-DRB1*01:01, HLA-DQA1*01:01, HLA-DQB1*05:01, and HLA-DPB1*23:01, were present only in the affected father and were not transmitted to the affected children, which may contribute to immunogenetic heterogeneity among patients. However, the functional relevance of these alleles needs validation.

### 3.3. In Silico Analysis of the PSMB10 c.56+1G>A Variant

**Figure 3:**
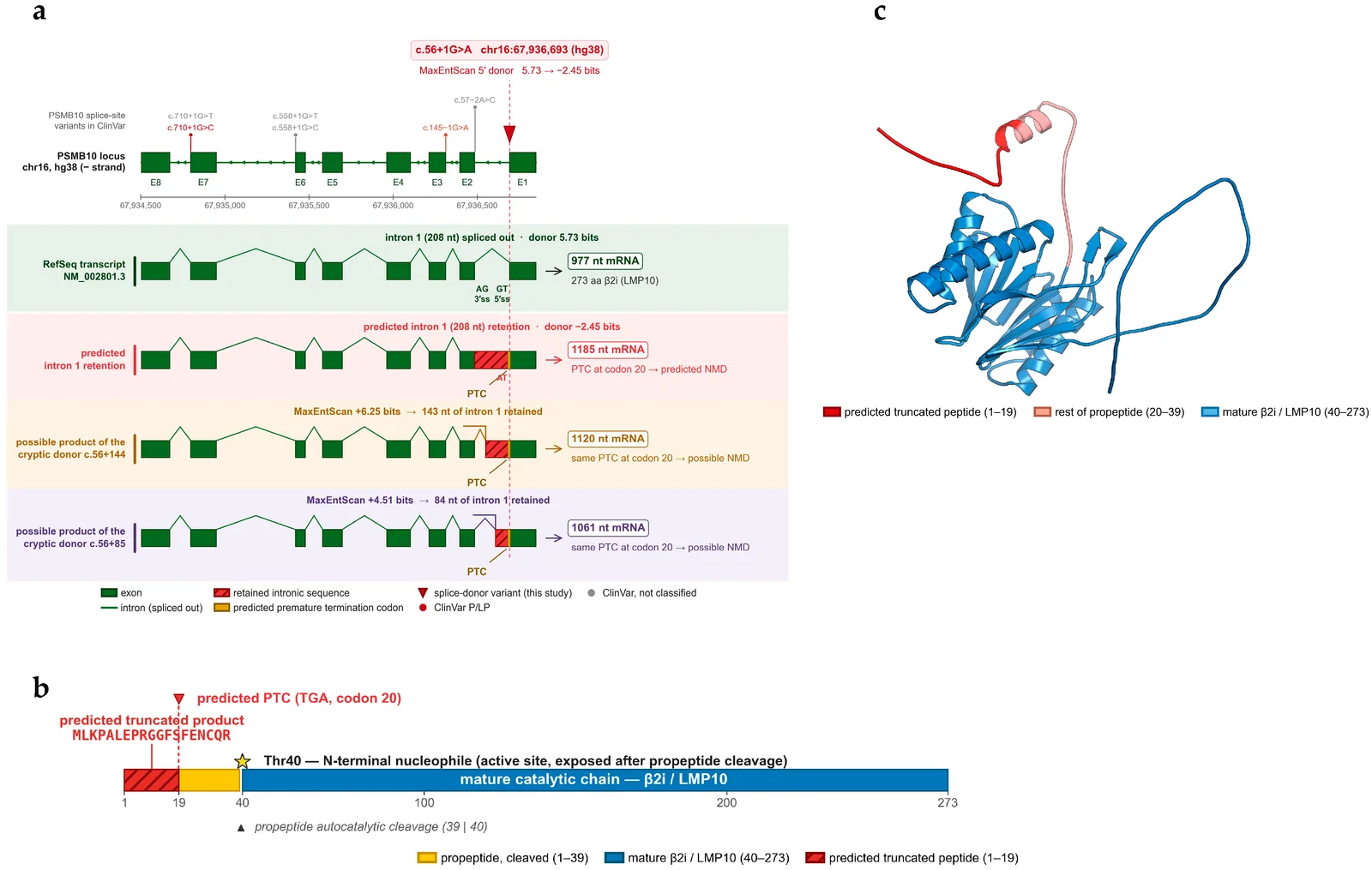
In silico analysis of *PSMB10* c.56+1G>A variant. (**a**) Predicted splicing consequence of *PSMB10* c.56+1G>A together with the MaxEntScan 5’ splice site scores of the canonical splicing, predicted main consequence (intron 1 retention), and two other possible cryptic donor sites. (**b**) Linear domain architecture of the *PSMB10* precursor. (**c**) Three-dimensional structure of the mature *PSMB10* catalytic subunit based on the human immunoproteasome 20S X-ray structure (PDB 6E5B, 2.77 Å).

Following variant identification and segregation analysis, we further performed in silico analyses to assess the consequences of the *PSMB10* c.56+1G>A variant at the transcript and protein level. In silico splicing analysis using *SpliceAI* demonstrated a Donor Loss with a Δ score of 1.00 at position +1 bp, predicting complete abolition of the canonical splice donor site at the intron 1 boundary, accompanied by an Acceptor Loss with a Δ score of 0.61 at −208 bp [29]. Pangolin independently corroborated these findings with a Splice Loss score of 0.78 at the same +1 bp position [30]. MaxEntScan [32] scored the reference intron 1 donor (AAG|GTGAAG) at 5.73 bits, whereas c.56+1G>A reduced it to −2.45 bits, an 8.18-bit (143%) loss, well beyond the 15% damaging threshold, predicting highly functional donor loss. No de novo splice donor site was created; cryptic sites at c.56 + 85 (4.51 bits) and c.56 + 144 (6.25 bits) remain possible but uncertain. The SAI-10k-calc algorithm [31] predicted intron 1 retention of a 208 bp coding sequence as the primary splicing consequence, resulting in a frameshift [30]. Complete or partial intron retention introduces the same premature TGA at codon 20 (Figure 3a). Supporting evolutionary constraint and functional impact, the variant received a CADD score of 34 and a PhyloP score of 8.85, reflecting high nucleotide conservation at this position [35] (Figure 3a). The disrupted donor is used by eight of eleven Ensembl *PSMB10* transcripts, including MANE Select NM_002801.4.

At the protein level, domain mapping showed that the canonical *PSMB10* precursor consists of an N-terminal propeptide followed by the mature β2i/LMP10 catalytic subunit (Figure 3b). This premature stop lies entirely within the autocatalytically excised propeptide region, upstream of the catalytic Thr40 nucleophile. Consequently, the predicted translated product in carriers is limited to a 19-residue N-terminal peptide, MLKPALEPRGGFSFENCQR, while the mature β2i/LMP10 catalytic β-sandwich domain is not produced (Figure 3b,c). Structural visualization further supported the prediction that the truncation occurs due to termination at codon 20; the mature β-sandwich fold and Thr40 active site are not produced (Figure 3c). Collectively, these computational analyses provide concordant evidence that c.56+1G>A disrupts the canonical *PSMB10* splice-donor site. Intron 1 retention represents the primary predicted aberrant transcript model and is predicted to result in a frameshift, generation of a premature termination codon, and consequent loss of normal protein function. However, computational analyses alone cannot exclude the possibility of additional aberrant splice products or establish their relative abundance in vivo.

### 3.4. Transcript-Level and Immunoproteasome-Related Gene Expression Analysis in Patient-Derived PBMCs

**Figure 4:**
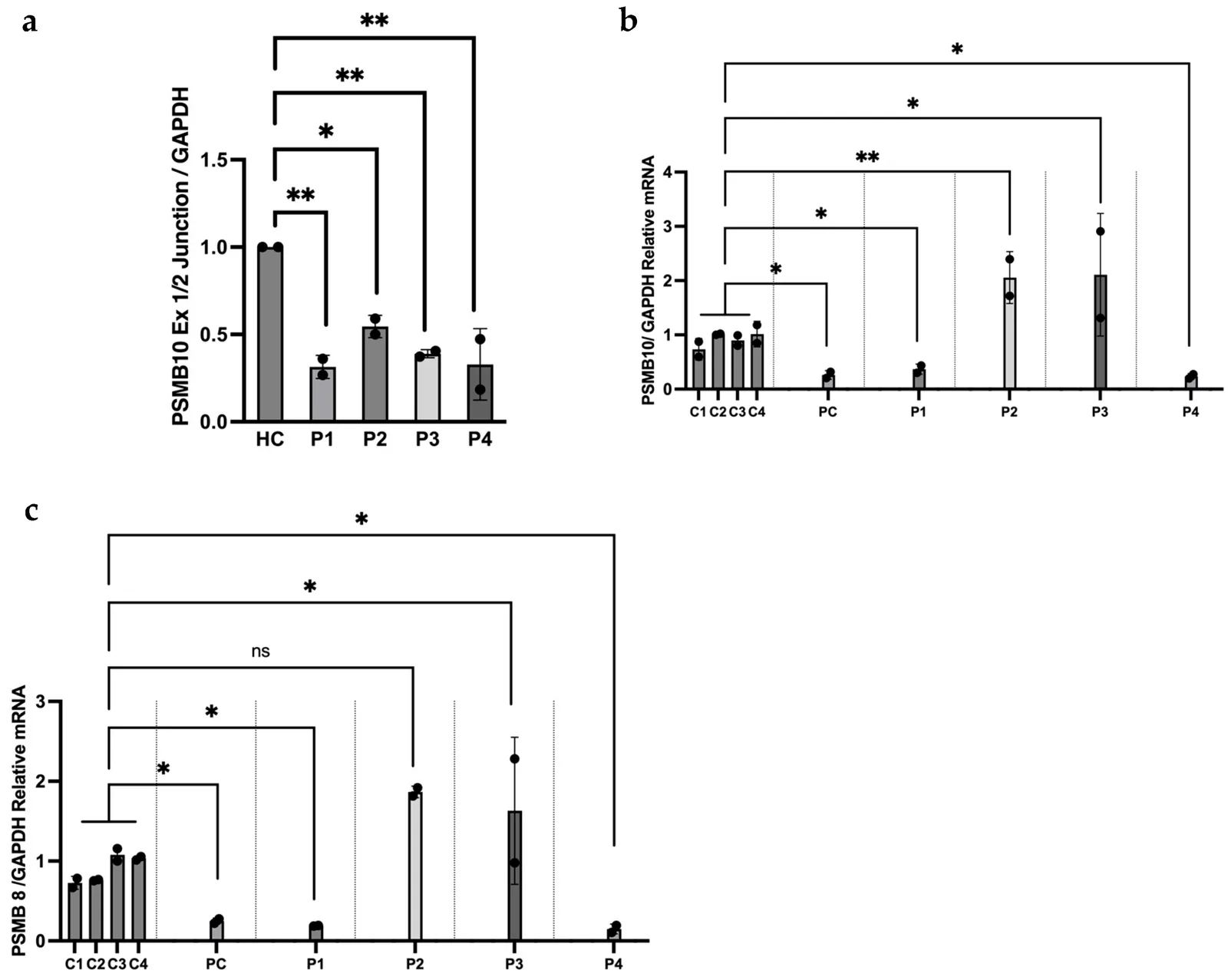
Assessment of *PSMB10/PSMB8* Expression in patients. (**a**–**c**) Total RNA was extracted from healthy and patient human PBMC cells and analyzed by qRT-PCR for (**a**) *PSMB10* exon1/2 splice junction, (**b**) *PSMB10*, and (**c**) *PSMB8*, with GAPDH used as the reference gene. Each bar represents one individual assayed in duplicate (two technical replicates). Relative fold change was calculated per subject: in (**a**) values are shown relative to a healthy control and statistical significance was determined by one-way ANOVA followed by Dunnett’s post hoc test, evaluating each subject against the baseline; in (**b**) and (**c**), values of each family member (*n* = 1) are shown relative to healthy control group (*n* = 4 independent individuals) and statistical significance was determined by nested *t*-test. Statistical details are provided in Methods, Statistics. Data are presented as mean ± SEM; *p*-value of <0.05 was considered statistically significant. * *p* < 0.05, ** *p* < 0.01; ns: not significant. HC: healthy control; C1–4: healthy control group; PC: unaffected patient control; P1–P4: affected individuals.

To provide experimental support for the predicted transcript-level consequence of the c.56+1G>A variant, we performed q-RT PCR to measure the abundance of the canonical *PSMB10* exon 1-exon 2 splice product in patient-derived peripheral blood mononuclear cells (PBMCs) isolated from the patients and a healthy control. Primers spanning the *PSMB10* exon 1–exon 2 junction were used, with each reaction assayed in duplicate (two technical replicates) using a single biological sample per individual. Given the limited number of individuals analyzed, the statistical comparisons presented below, including those in Section 3.5, should be regarded as exploratory rather than definitive. Affected individuals demonstrated reduced abundance of canonical exon1–exon2 product compared with the healthy control (Figure 3a and Figure 4a). This finding is consistent with the predicted effect of the c.56+1G>A variant on impaired formation or stability predictions of the normally spliced *PSMB10* transcript. However, the assay also does not determine the identity of the aberrant transcript generated following disruption of the canonical donor site. Accordingly, intron 1 retention and its predicted downstream consequences, including frameshift formation, generation of a premature termination codon, and predicted/possible nonsense-mediated mRNA decay (NMD), remain computationally inferred rather than experimentally demonstrated.

Immunoproteasomes play a critical role in maintaining cellular proteostasis and are functionally linked to autophagy pathways [36,37]. *PSMB10* encodes the β2i catalytic subunit of the immunoproteasome, a specialized form of the proteasome responsible for degradation of ubiquitinated proteins and regulation of immune and inflammatory responses [37].

To evaluate the impact of the identified *PSMB10* variant on cellular degradative pathways, we assessed the expression levels of *PSMB10* together with *PSMB8*, which encodes the β5i catalytic subunit of the immunoproteasome and is the best-characterized immunoproteasome gene in proteasome-associated autoinflammatory disease, within the patient cohort [18,38]. Analysis of each patient with respect to healthy controls (pool, *n* = 4) revealed that *PSMB10* mRNA levels were significantly upregulated (in more severely affected patients 2 and 3 (2 fold ** *p* < 0.01; 2.1 fold * *p* < 0.05 respectively), whereas others (mild phenotypes) (0.3, 0.2 fold respectively with * *p* < 0.05) and unaffected patient control was downregulated (0.2 fold, * *p* < 0.05.) (Figure 4b). The severity associated variaton and this opposite expression pattern, combined with the reduced abundance of canonical full-length *PSMB10* shown in Figure 4a, suggests that the change in total expression is not a global compensatory response to the splicing defect, but might instead correlate with the inflammatory status of the individuals.

Similar trend was observed for *PSMB8*, with increased expression levels for patient P2 (1.86 fold, * *p* < 0.05) and P3 (even if P3 could not reach to significance 1.6 fold, *p* ~ 0.08), whereas others exhibited lower expression levels with respect to healthy controls (P1: 0.24, P2: 0.19, P3: 0.15 fold with * *p* < 0.05) (Figure 4c). While the reason for the different expression levels of immunoproteasome subunits (*PSMB10* and *PSMB8*) among patients that carry the same variation remains unclear, our results might indicate that immunoproteasome-related pathways are differentially regulated among patients according to the severity of the phenotype.

### 3.5. Interferon-Related Gene Expression Analysis in Patient-Derived PBMCs

**Figure 5:**
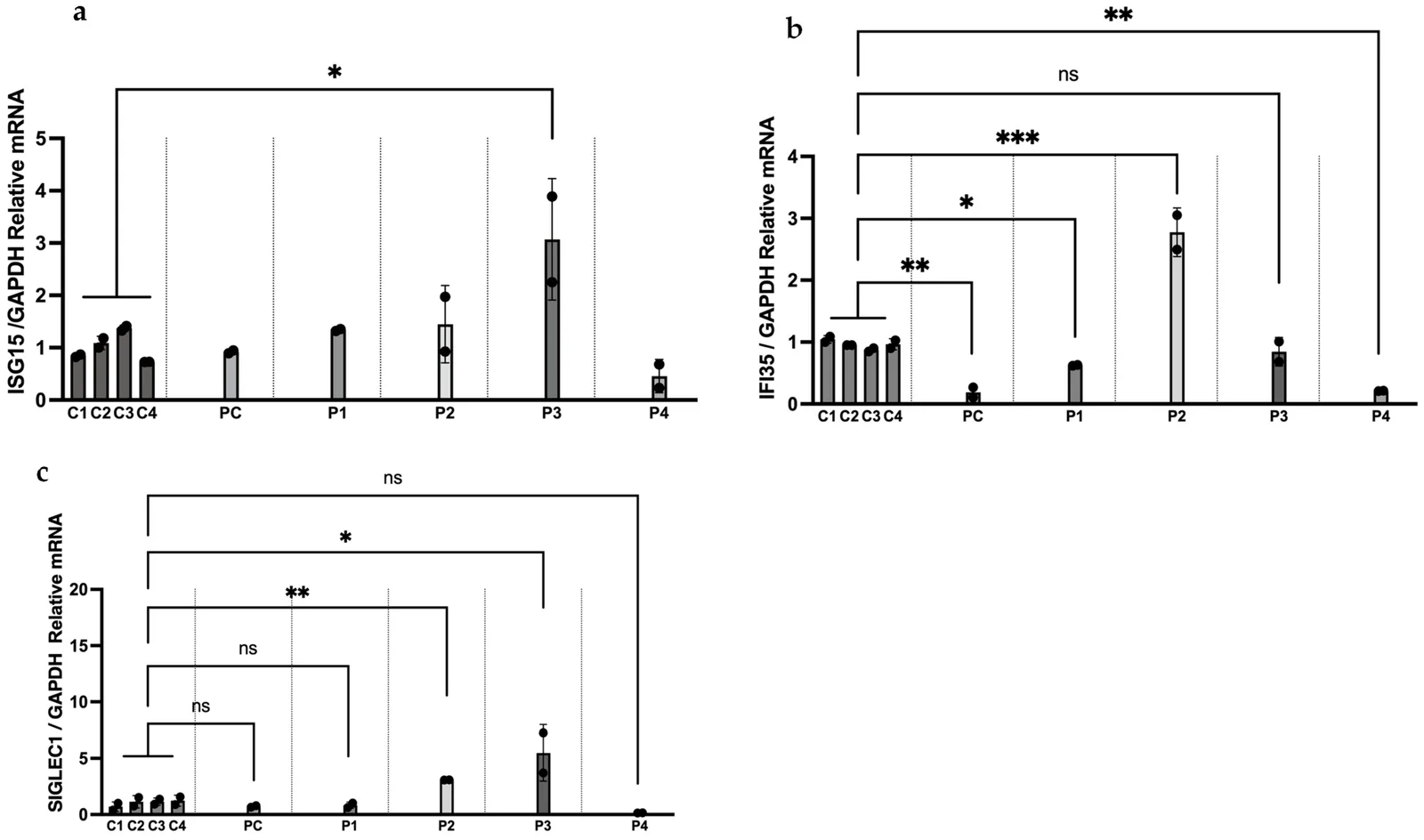
Interferon-related gene expression analysis. (**a**–**c**) Total RNA was extracted from healthy and patient human PBMC cells and analyzed by qRT-PCR for (**a**) *ISG15*, (**b**) *IFI35*, and (**c**) *SIGLEC1*, with GAPDH used as the reference gene. Each bar represents one individual assayed in duplicate (two technical replicates). Relative fold change was calculated per subject; values of each family member (*n* = 1) are shown relative to healthy control pool (*n* = 4 independent individuals). Each family member (*n* = 1) was compared with the control group (*n* = 4 individuals), and statistical significance was determined by nested *t*-test. Statistical details are provided in methods, statistics. Data are presented as mean ± SEM; *p* < 0.05 was considered statistically significant. * *p* < 0.05, ** *p* < 0.01, *** *p* < 0.001; ns: not significant. C1–4: healthy control group, PC: unaffected patient control, P1–P4: affected individuals.

Because proteasome dysfunction has been associated with type I interferon responses [18,20], we next explored whether patients carrying the *PSMB10* variant with q-RT PCR in patient-derived peripheral blood mononuclear cells (PBMCs) isolated from the patients (P1–P4), an unaffected patient control, and a healthy control group (*n* = 4). Primers specific for *ISG15*, *IFI35* and *SIGLEC1* were used, with each reaction assayed in duplicate (two technical replicates) using a single biological sample per individual. Analysis of IFN-signature-related genes revealed patient-specific patterns rather than a general and uniform interferonopathy-like response across all affected individuals, as shown in Figure 5.

*ISG15* expression was increased in P3 (Figure 5a), *IFI35* was elevated in P2 (Figure 5b), and *SIGLEC1* was increased in both P2 and P3 (Figure 5c). In contrast, P4 showed lower expression of all three genes, while P1 showed a similar overall reduction trend. The remaining evaluated IFN-related genes did not show consistent alterations across affected individuals. Overall, IFN-related gene expression varied among affected individuals, with the most pronounced changes observed in the clinically more severely affected individuals.

## 4. Discussion

In this study, we describe a two-generation Turkish family with a dominantly segregating autoinflammatory phenotype associated with a heterozygous splice-donor variant in *PSMB10* (c.56+1G>A). HLA typing revealed a paternally inherited haplotype that was shared by the affected father and all three affected offspring but absent in the unaffected mother (Table 2). This observation suggests that the affected individuals share an additional paternal immunogenetic background together with the shared *PSMB10* variant, raising the possibility that this haplotype may contribute to phenotypic variability as a modifier. However, given the small number of affected individuals and the absence of functional validation, any inference about a modifier effect on disease expression remains hypothesis-generating. Therefore, we note this shared immunogenetic background as an observation of potential interest, while acknowledging that its contribution to immune heterogeneity or clinical variability cannot be determined within a single pedigree. Further evaluation in additional unrelated *PSMB10*-associated families, together with functional studies, will be required before any causal modifier effect can be inferred.

Computational analyses consistently predicted disruption of the canonical *PSMB10* exon 1 donor site. Consistent with this prediction, the exon 1–exon 2 assay showed reduced abundance of the canonical splice product in variant carriers, providing transcript-level evidence of altered *PSMB10* processing. Although intron 1 retention is our primary predicted model, we identified two other potential but weaker cryptic donor sites; therefore, alternative splicing products cannot be completely excluded. Computational predictions suggest the introduction of an early PTC at around codon 20 and destruction of the aberrant transcripts by nonsense-mediated mRNA decay (NMD). However, NMD efficiency can be variable and context-dependent [39]. If those transcripts escape from the surveillance system, they can give rise to truncated proteins with dominant-negative or gain-of-function potential [40]. *PSMB10* variants have been reported to disrupt proteasome assembly [19]; this is structurally unlikely for the C56+1 G>A variant. The predicted product is 19 amino acid residue N terminal peptide, upstream of catalytic Thr40, which is too short to be incorporated into the 20S particle. Therefore, even if it escapes from NMD, continued translation would yield a severely truncated product that would be unlikely to assemble into an immune–proteasome complex.

We evaluated its possible association with immunoproteasome regulation and interferon-related responses. The family comprised five individuals: an unaffected mother who does not carry the variant; an affected father and a son who carry the variant, but both have a milder phenotype; and two sisters who carry the variant and have a more severe phenotype. Therefore, we assessed each affected individual separately because of the variable phenotypes. Our findings suggest that individuals with a more severe phenotype show more notable alterations in these pathways compared with mildly affected family members. Although the family was initially investigated in the context of an unclassified autoinflammatory phenotype overlapping with known monogenic autoinflammatory disorders, the present findings should be interpreted within the broader framework of proteasome-associated immune dysregulation rather than as evidence for a specific FMF-like *PSMB10*-associated syndrome. Previously reported *PSMB10*-associated disorders have primarily been discussed in relation to proteasome-associated autoinflammatory syndromes, particularly PRAAS, immunoproteasome dysfunction, immune dysregulation, and type I-interferon-related inflammation [17,18,20,41]. Therefore, our findings may expand the emerging spectrum of *PSMB10*-associated autoinflammatory disease, although the FMF-like features should be interpreted as clinical overlap rather than a defining characteristic of *PSMB10*-related disease.

This expansion is also geographic: FMF-like autoinflammatory phenotypes and the diagnostic challenges they pose, including *MEFV*-negative or atypical presentations requiring broader molecular evaluation, are well documented across Mediterranean-basin populations. FMF is highly prevalent among Armenian, Arab, and Jewish communities, and substantial case clusters have also been reported in Italian cohorts, reflecting shared genetic ancestry and the population-specific distribution of *MEFV* founder variants across the basin [3,4,5]. FMF-like phenotypes without biallelic pathogenic *MEFV* variants may reflect overlap syndromes or alternative monogenic etiologies [16], and family-based whole-exome sequencing strategies similar to ours have successfully resolved such unclassified presentations in FMF-prevalent populations: in Turkish families with clinical FMF, segregation-based exome analysis has identified pathogenic variants beyond the classical autoinflammatory disease panel, including a homozygous *MVK* mutation and a novel heterozygous *TNFRSF1A* mutation [13], and targeted next-generation sequencing panels have demonstrated diagnostic utility in the clinical suspicion of systemic autoinflammatory disease [12]. To our knowledge, no heterozygous *PSMB10* splice-site variant that segregates in an autosomal dominant pattern has been reported by other groups, neither in any of these populations nor in any autoinflammatory context; our preliminary findings were first presented at the 56th European Society of Human Genetics Conference [42]. The *PSMB10*-associated conditions described to date have largely presented as classical CANDLE/PRAAS, as proteasome-related immune dysregulation with T lymphopenia, or as de novo dominant disease, rather than as a dominantly segregating, FMF-overlapping presentation [17,18,19].

Catalytic subunits of immunoproteasomes are important for their role in degradation of ubiquitinylated proteins and consequently maintaining intracellular protein homeostasis [37,41]. *PSMB10* and *PSMB8* encode catalytic immunoproteasome subunits, β2i and β5i respectively; pathogenic variants in both genes have been linked to proteasome-associated autoinflammatory syndromes and to impaired protein homeostasis [17,18,38,41].

*PSMB10* and *PSMB8* expression differed markedly among affected family members, with higher total transcript levels observed in the more severely affected individuals and lower levels in the mildly affected individuals (Figure 4b,c). Although the mechanism underlying this difference remains unclear, this specific pattern might indicate a potential relationship between immunoproteasome-related gene expression and clinical severity.

While the exon 1/exon 2 junction assay shows a reduced abundance of the canonical splice product in variant carriers, overall *PSMB10* levels were elevated in severe cases; this reduction in exon 1–exon 2 signal in variant carriers suggests the disruption of normal *PSMB10* transcript processing but does not directly identify the aberrant RNA species. The phenotypic difference among patients suggests that a global cell-autonomous compensatory mechanism is unlikely. Instead, the increased expression of immunoproteasome-associated genes in more severe patients may reflect an inflammation-driven transcriptional response associated with impaired canonical splicing and altered proteasome biology. Thus, the increased total expression of *PSMB10* does not necessarily indicate restored transcription; rather, it may reflect a combination of altered transcription and disease-associated transcriptional regulation. Collectively, whereas the reduced expression observed in mildly affected variant carriers suggests that the *PSMB10* variant alone may not be sufficient to drive immunoproteasome upregulation, additional factors, such as disease activity, cellular inflammatory state, or modifier genetic background, may influence the transcriptional response, which requires further investigation [18,20,37]. Therefore, these expression findings should be interpreted as an observed association with clinical severity rather than direct evidence of altered proteasome function.

Proteasome-associated autoinflammatory diseases link impaired protein clearance with immune dysregulation and type I interferon-related inflammatory responses [18,20]. However, the clinical and molecular consequences of proteasome gene variants may be heterogeneous and limited, and context-dependent interferon-stimulated gene expression can be observed in some cases [43,44,45]. In this context, previous studies suggest that incomplete protein clearance and proteotoxic stress may engage cellular stress-response and innate immune pathways, providing a possible link between proteasome impairment and selective IFN-related transcriptional changes without necessarily inducing a generalized interferonopathy-like response [46,47]. These observations provided a rationale for evaluating IFN-related gene expression changes in this family.

Evaluation of IFN-related genes revealed limited but notable and context-dependent differences, mostly in the more severely affected patients. *SIGLEC1*, *IFI35*, and *ISG15* showed an overall reduced expression pattern in milder phenotypes, including significant downregulation in one patient. Severely affected patients showed concomitant upregulation of *ISG15*, and *IFI35* expression levels were elevated in one individual of each, and *SIGLEC1* expression levels were elevated in both. This distribution might suggest heterogeneous activation of inflammatory pathways rather than a generalized interferon response across all affected individuals. Although there is some activation in certain signature-related genes, it is not strong (not significant) and/or consistent among affected individuals. Given the small sample size and the limited number of IFN-related genes showing expression differences, these data should be interpreted cautiously and do not establish a causal relationship between IFN-related gene expression and clinical severity. Furthermore, although *SIGLEC1* has been reported as a useful marker of type I IFN activity [48], the present data do not directly support a functional hierarchy among *SIGLEC1*, *IFI35*, and *ISG15*. Therefore, these changes should be interpreted as exploratory molecular findings that may arise secondary to broader *PSMB10*-associated immunoproteasome dysregulation, patient-specific inflammatory status, and possible epistatic interactions involving unknown genetic or epigenetic modifiers, rather than as direct evidence of a primary interferonopathy [18,20].

The main strength of the present study is that, even though the study was performed in a single family, the presence of multiple affected individuals across two generations, together with an unaffected family member, provided a valuable opportunity for segregation analysis and intrafamilial comparison of molecular findings in relation to clinical severity. In this context, evaluation of immunoproteasome-related gene expression, IFN-related markers, and the shared immunogenetic background revealed molecular patterns that appeared more pronounced in the more severely affected individuals. Taken together, this two-generation family structure is particularly informative, despite the limitations, in the context of a rare disorder, as it allows genetic findings and molecular evaluation across several affected relatives.

Several limitations should be considered when interpreting these findings. The molecular analysis was based on a limited number of individuals, with expression studies performed using a single biological sample per subject, assayed in duplicate (two technical replicates). Consequently, this restricts the generalizability of the results. The statistical analyses should be interpreted cautiously, and the observed molecular differences should be regarded as exploratory findings that require validation in additional patients or families. In addition, the aberrant RNA species generated by the splice-site variant was not directly characterized; therefore, the predicted intron 1 retention remains computationally inferred. Moreover, significant expression differences within the IFN-related pathway were observed in only a limited number of genes and appeared to parallel clinical severity; therefore, these findings should not be regarded as evidence of a comprehensive interferon signature. Broader functional studies in larger cohorts, preferably including unrelated families or patients, will be important to extend these findings and clarify the role of *PSMB10* in proteasome-associated autoinflammatory disease. Finally, the potential contribution of the shared paternal HLA background or other modifier factors cannot be determined from this family alone and remains a hypothesis for future investigation.

## 5. Conclusions

Overall, our findings suggest that the identified *PSMB10* c.56+1G>A variant is associated with immunoproteasome-related transcript levels in patient-derived PBMCs, with the most pronounced molecular changes occurring in clinically more severely affected individuals. Together with familial segregation, concordant in silico prediction of splice-donor disruption, and reduced abundance of the canonical exon 1–exon 2 transcript, these findings support the variant as a strong candidate contributor to the autoinflammatory phenotype observed in this family.

Although these molecular alterations broadly paralleled the intrafamilial clinical heterogeneity, we emphasize that the present data are exploratory and do not establish a causal genotype–phenotype relationship. Limited and heterogeneous IFN-related gene expression changes were also observed, particularly in clinically more severe individuals. Importantly, the intrafamilial variability observed in this family suggests that autoinflammatory diseases that appear to follow a monogenic inheritance pattern may still involve a more complex genetic architecture, in which modifier variants, immunogenetic background, disease activity, and pathway-level interactions influence clinical severity and molecular responses. Further studies in additional families and larger patient cohorts, together with modifier-gene analyses and mechanistic functional assays, including direct proteasome activity assays, β2i protein-level validation, and broader transcriptomic profiling, will be important to validate these findings and conclusively establish how the *PSMB10* c.56+1G>A variant contributes to proteasome-associated autoinflammatory disease.

## Figures and Tables

**Table 1:** Demographics and multi-system clinical phenotype of five-member Turkish family segregating the immunoproteasome *PSMB10* splice-donor variant c.56+1G>A. The asterisk (*) indicates affected individuals; PC = mother/unaffected family control. “+” = feature explicitly documented as present; “−” = feature explicitly documented as absent; “^†^” = intermittent elevation; “NR” = not reported (no inference); ”dx” = Diagnosis; “—”, not applicable. PRUNED CONVENTION: phenotype rows for which no family member is documented as present have been removed for clarity (3 rows: fatigue/malaise, Interstitial Lung Disease (ILD) screening, Chronic Obstructive Pulmonary Disease (COPD)-targeted bronchodilator). Demographics and genetic-test rows with results are retained even where some individuals are NR. The table is strictly derived from the primary clinical source records and annotated with verified Human Phenotype Ontology (HPO) identifiers [27].

Characteristic (HPO ID)	P1 *	PC	P2 *	P3 *	P4 *
Demographics					
Sex	M	F	F	F	M
Age at first evaluation (years)	*NR*	*NR*	23	24	*NR*
Age at last evaluation (years)	*NR*	*NR*	27	28	*NR*
Height (m)	*NR*	*NR*	1.60	1.56	*NR*
Weight (kg)	*NR*	*NR*	92	98	*NR*
Body Mass Index (BMI, kg/m^2^)	*NR*	*NR*	35.9	40.3	*NR*
Geographic origin	Kastamonu	Sivas	Kastamonu	Kastamonu	*NR*
Consanguinity	No	No	No	No	No
Age at symptom onset (years)	*NR*	*NR*	+ (7–8)	+ (7–8)	*NR*
First medical evaluation (year)	*NR*	*NR*	+ (2015)	+ (2015)	*NR*
Lifestyle Factors					
Smoking History	*NR*	*NR*	No	No	*NR*
Alcohol consumption	*NR*	*NR*	No	No	*NR*
Constitutional symptoms					
Recurrent fever (HP:0001954)	*NR*	*NR*	+	+	*NR*
Cachexia/severe weight loss (HP:0004326)	*NR*	*NR*	+ (35 kg/5 months)	*NR*	*NR*
Mucocutaneous involvement					
Malar rash (HP:0025300)	*NR*	*NR*	+	+	*NR*
Cutaneous photosensitivity (HP:0000992)	*NR*	*NR*	*NR*	+	*NR*
Recurrent aphthous stomatitis (HP:0011463)	*NR*	*NR*	+	+	*NR*
Alopecia (HP:0002293)	*NR*	*NR*	+	+	*NR*
Erythema nodosum (HP:0012219)	*NR*	*NR*	+ (childhood)	+ (childhood)	*NR*
Sicca syndrome (Sjögren spectrum)					
Keratoconjunctivitis sicca, Schirmer-positive (HP:0001097)	*NR*	*NR*	+	+	*NR*
Xerostomia (HP:0000217)	*NR*	*NR*	+	+	*NR*
Dysphagia (HP:0002015)	*NR*	*NR*	*NR*	+	*NR*
Vasculopathy					
Raynaud phenomenon (HP:0030880)	*NR*	*NR*	+	+	*NR*
Musculoskeletal involvement					
Polyarthritis (HP:0005764) ± joint stiffness (HP:0001387)	*NR*	*NR*	+ (R ankle, R wrist, MCP)	+ (knee, L wrist, MCP)	*NR*
Inflammatory low back pain (HP:0003418)	*NR*	*NR*	+	+	*NR*
Arthralgia (HP:0002829)	*NR*	*NR*	+ (childhood)	+ (childhood)	*NR*
Cardiovascular and pulmonary involvement					
Vascular occlusive disease	+	+	*NR*	*NR*	*NR*
Arterial hypertension (HP:0000822)	+	+	*NR*	*NR*	+ (Holter monitoring)
Cardiac arrhythmia evaluation (HP:0011675)	*NR*	*NR*	*NR*	*NR*	+ (Holter monitoring)
Chronic obstructive pulmonary disease (COPD) (HP:0006510)	+	*NR*	*NR*	*NR*	*NR*
Pleurisy/pleuritic chest pain (HP:0001875)	*NR*	*NR*	+	+	*NR*
Dyspnea (HP:0002094)	*NR*	*NR*	+	+	*NR*
Gastrointestinal involvement					
Recurrent abdominal pain (HP:0002574)	*NR*	*NR*	+ (1–2 flares/month)	+	+ (intermittent)
Chronic diarrhea (HP:0002028)	*NR*	*NR*	+ (1–month, 15–20 stools/day)	+ (2–3 day)	+ (intermittent)
Nausea (HP:0002018)	*NR*	*NR*	+	+	*NR*
Ocular (inflammatory and structural)					
Uveitis (HP:0000554)—suspected	*NR*	*NR*	*NR*	+	*NR*
Congenital cataract (HP:0000519)	*NR*	*NR*	+ (operated)	*NR*	*NR*
Neurological involvement					
Generalized epilepsy (HP:0002197)	*NR*	*NR*	*NR*	+ (3-years)	*NR*
Syncope (HP:0001279)—pain-triggered	*NR*	*NR*	*NR*	+	*NR*
Endocrine and metabolic					
Obesity	*NR*	*NR*	+ (BMI 35.9 kg/m^2)^	+ (BMI 40.3 kg/m^2)^	*NR*
Insulin resistance	*NR*	*NR*	+	*NR*	*NR*
Polycystic Ovary syndrome (PCOS)	*-*	*NR*	+	*NR*	*-*
Diabetes Mellitus	+	*NR*	*NR*	*NR*	*NR*
Primary hypothyroidism (HP:0000821)	*NR*	+	+ (post-RAI)	+	*NR*
Differentiated thyroid carcinoma (HP:0002890)	*NR*	*NR*	+ (total thyroidectomy + RAI)	*NR*	*NR*
Metabolic and biochemical laboratory findings					
Total cholesterol (mg/dL)	*NR*	*NR*	198.2	185	*NR*
LDL cholesterol (mg/dL)	*NR*	*NR*	143.7	110	*NR*
HDL cholesterol (mg/dL)	*NR*	*NR*	52.2	51	*NR*
Triglycerides (mg/dL)	*NR*	*NR*	77.5	146	*NR*
Glucose (mg/dL)	*NR*	*NR*	68	76	*NR*
Creatinine (mg/dL)	*NR*	*NR*	0.56	0.78	*NR*
AST (IU/L)	*NR*	*NR*	65 ^†^	17.6	*NR*
ALT (IU/L)	*NR*	*NR*	104 ^†^	15	*NR*
Reproductive/gynecological					
Recurrent ovarian torsion (HP:0008733)	*—*	*NR*	+ (2017-19)	*NR*	*—*
Renal involvement					
Proteinuria (HP:0000093)	*NR*	*NR*	*NR*	+ (2017)	*NR*
Infectious history					
Mycobacterial infection (HP:0032279), renal localization	*NR*	*NR*	*NR*	+ (renal TBC)	*NR*
PPD-positive/latent TBC (HP:0032279)	*NR*	*NR*	+ (latent)	+ (active)	*NR*
Autoantibodies					
Antinuclear antibody (ANA) positivity (HP:0003493)	*NR*	*NR*	+	+	*NR*
Working clinical diagnoses (chronological)					
Henoch-Schönlein purpura (HSP) (HP:0010748)—suspected, 2015	*NR*	*NR*	*NR*	+	*NR*
Vasculitis (HP:0002633)—initial differential	*NR*	*NR*	*NR*	+	*NR*
Familial Mediterranean fever (clinical dx; OMIM #249100)	*NR*	*NR*	+	+	*NR*
Juvenile idiopathic/rheumatoid arthritis (HP:0030308)	*NR*	*NR*	+ (differential)	+ (RA dx)	*NR*
Systemic lupus erythematosus (HP:0002725)	*NR*	*NR*	+	+	*NR*
PRAAS5 (*PSMB10* c.56+1G>A AD; OMIM #619175)	+	−	+	+	+
Current and historical pharmacotherapy					
Rituximab—Mabthera^®^	*NR*	*NR*	+	*NR*	*NR*
Hydroxychloroquine—Plaquenil^®^	*NR*	*NR*	+	*NR*	*NR*
Methotrexate (MTX)	*NR*	*NR*	+	*NR*	*NR*
Abatacept—Orencia^®^	*NR*	*NR*	+	*NR*	*NR*
Azathioprine—Imuran^®^	*NR*	*NR*	+	*NR*	*NR*
Mycophenolate mofetil—CellCept^®^	*NR*	*NR*	+	*NR*	*NR*
Tacrolimus	*NR*	*NR*	+	*NR*	*NR*
Colchicine	*NR*	*NR*	+ (intermittent)	+ (offered; self-discontinued)	*NR*
Sulfasalazine—Salozopyrin^®^ (historical)	*NR*	*NR*	+	+	*NR*
Indomethacin—Endol^®^ (historical)	*NR*	*NR*	+	+	*NR*
Anti-tuberculosis therapy	*NR*	*NR*	+ (INH irregular)	+ (R + E + H × 1.5 years)	*NR*
Levothyroxine replacement	*NR*	+	+(150 μg/day)	+ (150 μg/day)	*NR*
Antihypertensive therapy	+	+	*NR*	*NR*	+ (workup)
Levetiracetam (Keppra^®^)—historical	*NR*	*NR*	*NR*	+ (self-discontinued)	*NR*
Most Recent Medication					
Upadacitinib	*NR*	*NR*	+ (15 mg/day)	+ (15 mg/day)	*NR*
Quetiapine	*NR*	*NR*	*NR*	+ (300 mg/day)	*NR*
Venlafaxine	*NR*	*NR*	*NR*	+ (150 mg/day)	*NR*
Orlistat	*NR*	*NR*	*NR*	+ (120 mg/day)	*NR*
Surgical and procedural history					
Congenital cataract surgery	*NR*	*NR*	+	*NR*	*NR*
Total thyroidectomy + RAI	*NR*	*NR*	+	*NR*	*NR*
Ovarian de-torsion surgery (2017-19)	*—*	*NR*	+ (recurrent)	*NR*	*—*
Hospitalization for inflammatory exacerbations	*NR*	*NR*	+ (multiple)	+ (multiple)	*NR*

**Table 2:** HLA Allele genotypes of a five-member Turkish family. P1 affected father, P2–P4 affected children, PC unaffected mother. Alleles shared among affected individuals are given in purple. Alleles that are present only in affected father are given in green. Alleles that are shared among unaffected mother and patients are not highlighted.

Locus	Class	PC-A1	PC-A2	P1-A1	P1-A2	P2-A1	P2-A2	P3-A1	P3-A2	P4-A1	P4-A2
**HLA-B**	**Class I**	B*07:02:01	B*18:01:01	B*44:02:01	B*35:01:01	B*18:01:01	B*44:02:01	B*07:02:01	B*44:02:01	B*18:01:01	B*44:02:01
**HLA-C**	**Class I**	C*12:03:01	C*07:02:01	C*16:04:01	C*04:01:01	C*16:04:01	C*12:03:01	C*07:02:01	C*16:04:01	C*16:04:01	C*12:03:01
**HLA-G**	**Class Ib**	G*01:01:01	-	G*01:01:01	G*01:04:01	G*01:04:01	G*01:01:01	G*01:01:01	G*01:04:01	G*01:01:01	G*01:04:01
**DRB1**	**Class II—DR**	DRB1*15:01:01	DRB1*11:04:01	DRB1*11:04:01	DRB1*01:01:01	DRB1*11:04:01	-	DRB1*15:01:01	DRB1*11:04:01	DRB1*11:04:01	-
**DQA1**	**Class II—DQ**	DQA1*01:02:01	DQA1*05:05:01	DQA1*01:01:01	DQA1*05:05:01	DQA1*05:05:01	-	DQA1*01:02:01	DQA1*05:05:01	DQA1*05:05:01	-
**DQB1**	**Class II—DQ**	DQB1*03:01:01	DQB1*06:02:01	DQB1*05:01:01	DQB1*03:01:01	DQB1*03:01:01	-	DQB1*06:02:01	DQB1*03:01:01	DQB1*03:01:01	-
**DPB1**	**Class II—DP**	DPB1*04:02:01	DPB1*04:01:01	DPB1*23:01	DPB1*02:01:02	DPB1*04:02:01	DPB1*02:01:02	DPB1*04:01:01	DPB1*02:01:02	DPB1*04:02:01	DPB1*02:01:02
**DMA**	**Class II—DM**	DMA*01:01:01	-	DMA*01:01:01	DMA*01:02	DMA*01:01:01	DMA*01:02	DMA*01:01:01	DMA*01:02	DMA*01:01:01	DMA*01:02

## Data Availability

The clinically relevant variant identified in this study has been deposited in the NCBI Linvar database under the variant accession number VCV003780496.7 (Variation ID: 3780496; with submitter accession number SCV007596971.1). The record is publicly available and can be directly accessed via the following web link: https://www.ncbi.nlm.nih.gov/clinvar/variation/3780496/ with the accession date of 6 June 2026.

## Abbreviations

The following abbreviations are used in this manuscript:

CAMPS	CARD14-mediated psoriasis
CAPS	Cryopyrin-associated periodic syndromes
CANDLE/PRAAS	Chronic Atypical Neutrophilic Dermatosis with Lipodystrophy and Elevated Temperature/Proteasome-associated Autoinflammatory Syndrome
DIRA	Deficiency of the Interleukin-1 Receptor Antagonist
DITRA	Deficiency of the Interleukin-36 Receptor Antagonist
EOS	Early-onset sarcoidosis
FMF	Familial Mediterranean Fever
SLE	Systemic Lupus Erythematosus
HSP	Henoch-Schonlein Purpura
ILS	Interstitial Lung Disease
COPD	Chronic Obstructive Pulmonary Disease
RAI	Radioactive Iodine Therapy
HC	Healthy Control
IFI35	Interferon-Induced Protein 35
IFN	Interferon
ISG15	Interferon-Stimulated Gene 15
MEFV	Mediterranean Fever Gene
MKD-HIDS	Mevalonate kinase deficiency/Hyperimmunoglobulin D syndrome
NLRP12AD	NLRP12-associated autoinflammatory disorder
PC	Patient Control (unaffected family member)
PAPA	Pyogenic arthritis, pyoderma gangrenosum and acne syndrome
PBMC	Peripheral Blood Mononuclear Cells
PSMB8	Proteasome 20S subunit beta 8
PSMB10	Proteasome 20S subunit beta 10
TRAPS	Tumor Necrosis Factor receptor-associated periodic syndrome
WES	Whole-exome sequencing
